# Validity and Reliability of the Filipino Version of the Kessler Psychological Distress Scale

**DOI:** 10.7759/cureus.85657

**Published:** 2025-06-09

**Authors:** Shodai Sunagozaka, Takashi Izutsu, Eizaburo Tanaka, Nicanor L Echavez, Paul Kenneth M Benavidez, Atsuro Tsutsumi

**Affiliations:** 1 Graduate School of Human and Socio-Environmental Studies, Kanazawa University, Kanazawa, JPN; 2 Graduate School of Agricultural and Life Sciences, The University of Tokyo, Tokyo, JPN; 3 College of Arts and Sciences, The University of Tokyo, Tokyo, JPN; 4 Community-Based Mental Health Clinic, Muntinlupa City Health Office, Muntinlupa, PHL; 5 Community-Based Mental Health Clinic, Muntinlupa City Health Office, Munthinlupa, PHL; 6 Institute of Transdisciplinary Sciences for Innovation, Kanazawa University, Kanazawa, JPN

**Keywords:** depression, k6, mental health, philippine, psychological distress

## Abstract

Introduction: Kessler Psychological Distress Scale (K6) is a self-administered and short screening instrument for non-specific psychological distress and is used internationally in epidemiological studies because of its good psychometric properties. K6 can be feasibly used to screen for mental health problems in settings with limited mental health professionals, such as in low- and middle-income countries. This study aims to examine the psychometric validity, construct validity, and internal consistency of the Filipino version of K6.

Methods: The participants were recruited from the community in Muntinlupa City, Philippines, and from patients in a psychiatric unit run by the local government in Muntinlupa. A structured questionnaire was used to collect data from both groups. The questionnaire assessed sociodemographic characteristics and included the K6 scale. For outpatients, in addition to the items used for community members, Patient Health Questionnaire-9 (PHQ-9), World Health Organization-Five Well-Being Index (WHO-5), and World Health Organization Quality of Life - BREF (WHOQOL-BREF) were employed to examine construct validity. Depression severity and diagnosis were determined by a psychiatrist or a resident doctor based on an unstructured clinical interview with three options: mild, moderate, and severe, and diagnostic criteria, respectively. Internal consistency and psychometric validity were assessed using Cronbach’s α and an unpaired t-test for the total K6 score between patients assessed as having mild depression and those with moderate or severe depression, respectively. K6 scores of community people and patients from the psychiatric unit were compared using a Mann-Whitney U test.

Results: In total, 95 people from the psychiatric unit (27 male patients, 77 female patients, and one gender-diverse) and 405 people from the community (178 male participants, 226 female participants, and one gender-diverse) participated in the study. Cronbach’s α of K6 was 0.88. Patients in the psychiatric unit had higher K6 total scores (median 14.00) than the participants from the community (median 3.00). Coefficient correlation analysis showed that K6 was strongly and positively correlated with PHQ-9 (*r* = 0.74, *p *< 0.01) and moderately and negatively correlated with WHO-5 (*r* = -0.51, *p* <0.01) and Psychosocial health in WHOQOL-BREF (*r* = -0.59, *p *< 0.01). The optimal cutoff points for moderate and serious psychological distress were 6/7 and 11/12, respectively, based on the distribution of scores. Patients assessed as having moderate or severe depression had a statistically higher K6 score (Mean 15.60) than those assessed as having mild depression (Mean 12.96).

Conclusion: The Filipino version of K6 is appropriate for measuring psychological distress in clinical and community settings. The Filipino version of K6, including cutoff points, will be a useful tool in the local context in practice and can be used as a measurement tool in studies to promote mental health in various settings.

## Introduction

Short screening instruments for detecting major mental disorders and psychological distress are currently used not only in psychiatric settings, but also in the community setting [1,2]. For example, it is used in the study on the mental health of people living with HIV and epidemiological mental health surveys and to assess the effect of education on mental health in schools [3-5].

A standardized instrument such as the Health Opinion Survey for quantifying mental health status and a non-specific distress scale were developed in the 1950s for epidemiological surveys [6]. Later, interview-based tests by experts in psychiatry, such as the Diagnostic Interview Schedule (DIS) and Mini-International Neuropsychiatric Interview [7,8], were developed. Interview-based tests have good psychometric properties; however, time and professional human resources are required to conduct them. Self-administered and short instruments are easy to use regardless of mental health-related human resources and are an effective measure in promoting mental health in the community. Focusing on the severity of mental health issues, including health-related quality of life, is a global trend in mental health research [9,10]. 

Several mental health screening instruments are available, such as Patient Health Questionnaire-9 (PHQ-9), General Health Questionnaire (GHQ), and Kessler Psychological Distress Scale (K6) [10-12]. Among these, K6 has strong psychometric properties and can also identify nonspecific psychological distress with a unidimensional structure and brief items (six items) [13].

K6 is a self-administered instrument for psychological distress in the community [10]. Psychological distress is a comprehensive term for multiple common psychological conditions, including subclinical and clinical symptoms [14]. K6 has been used in various populations, including people with various mental health disorders, and translated into various languages, such as Arabic, French, Japanese, and Vietnamese [15-19].

There is a strong demand to promote mental health in low- and middle-income countries (LMICs) [20]. For instance, the prevalence rate of major mental disorders does not differ statistically according to each country's income level, but only 6% of studies on mental health were published in LMICs [21-23]. The World Health Organization (WHO) also indicated a lack of data on mental health, especially in LMICs [24].

In the Philippines, the human resources for mental health are limited. There are 0.518 psychiatrists and 0.885 psychologists per 10000 of the population in the Philippines as of 2016 [25]. The number of mental health professionals is lower than that in other Western Pacific Rim countries such as Indonesia and Malaysia [26]. The 2005 World Health Survey, initiated by the WHO, reported that 14.5% of participants had a diagnosis of depression [26].

In addition, low awareness, stigma, and a strong relationship with a family member hinder people with mental health problems from accessing healthcare services [27-29]. Therefore, a validated instrument to screen mental health problems is important for people to know the needs for healthcare in their mental health, regardless of mental health literacy. Moreover, as the number of mental health professionals is limited, self-administered instruments for non-specific psychological distress can be utilized in primary care settings and reference for examination. In academic perspectives, validation of instruments on mental health will be expected to result in further study related to mental health in the Philippines.

Therefore, this study aims to examine the psychometric validity, construct validity, and internal consistency of the Filipino version of K6 in clinical and community settings.

## Materials and methods

Study setting

This study was conducted in Muntinlupa City, which is located in the southern part of the Manila Area and has a population of 543,445 people as of 2020 [30]. The Philippines is categorized as a lower-middle-income country by the World Bank [31]. The study setting was a community and psychiatric unit run by the City of Muntinlupa. The psychiatric unit is located in a community-based health center. The consultation fee in the center is free of charge.

Participants

This study is a cross-sectional validation study of the Filipino version of K6 with a study sample of participants from the psychiatric unit and from the community. Participants were recruited from the psychiatric unit of a community-based health center in Muntinlupa, the Philippines. In addition, participants in the community sample were recruited from among those registered in the official resident registry of Muntinlupa City. Data were collected from October to December 2024 and in December 2019, respectively. COVID-19 influenced the gap in data collection periods.

For participants from the psychiatric unit, participants were recruited consecutively on clinic days, without prior announcement, to minimize selection bias using the convenience sampling method. The participants received 500 Philippine pesos (8.57 USD, as of January 29, 2025, according to Morningstar, Inc.) as compensation for the long study period.

For participants from the community, we randomly approached 800 people who are over 18 years old in Muntinlupa based on the official resident registry using a randomized sampling method. Gender, age, and district of living were not stratified in the randomized process. The city government requested the selected participants to participate in health promotion events and data collection was conducted in the event. Of them, 405 from all districts in Muntinlupa participated in the study. They received 300 pesos (5.14 USD, as of January 29, 2025, according to Morningstar, Inc.) as compensation for participation in the study.

The inclusion criterion for community people was age over 18 years, and no exclusion criteria were set, such as illiteracy or disability. Regarding participants from the psychiatric unit, patients who had not been diagnosed yet based on the Diagnostic and Statistical Manual of Mental Disorders, Fifth Edition, Text Revision (DSM-5-TR) [32] were excluded.

The required sample size was decided by an a priori power analysis using G*Power 3.1 (Heinrich-Heine-Universitt Dsseldorf, Dsseldorf, Germany). Assuming a two-sided Mann-Whitney U test on K6 score between patients in a psychiatric unit, and community people, a medium effect size (*r* = 0.24, equivalent to Cohen’s d = 0.5), a significance level of α = 0.05, and a power of 0.80, the analysis indicated that a sample size of 67 participants per group would be necessary to detect a statistically significant difference. However, it was expected that the certain number of community people who were invited to participate in the study as part of a health promotion event would deny participation, and more people than the required sample size were approached in this study.

Questionnaires

A structured questionnaire was administered in this study. The questionnaire items used in this study for patients in the psychiatric unit partly differed from those used for community members. Sociodemographic characteristics, such as gender, years of education, number of family members living together, and K6, were assessed in both groups, and other items were employed in patients in the psychiatric unit.

K6 has six instruments with 0-4 response options, and “all of the time” is coded as 4, and “none of the time” is coded as 0. The total score ranges from 0 to 24, and a higher score indicates higher psychological distress. The original version of K6 (AUC Index = 0.94) indicates that 12/13 is the optimal cutoff point for serious mental illness [33]. K6 was translated into the Filipino language using an academic normative method. First, the original English version was translated into Filipino separately by two health workers who frequently spoke both English and Filipino. The Filipino version was then back-translated by two different people. Two sets of back-translated documents were checked by researchers in public health and mental health, and clinicians in the Philippines with necessary revisions.

The Filipino version of PHQ-9 was administered to participants from the psychiatric unit. PHQ-9 was originally developed for patients in primary care clinics and obstetrics clinics in the USA [11]. The Filipino version was developed by Mapi Research Institute of Pfizer and is freely available online [34]. PHQ-9 is a four-point Likert scale with nine items and a standardized instrument for common mental disorders. It is widely used in primary care settings to screen for depression [35]. The total score ranges from 0 to 27, and a higher score indicates worse mental health conditions. The Filipino version of PHQ-9 is standardized among Filipino migrant domestic workers in Macao [36].

Additionally, the Filipino version of The World Health Organization-Five Well-Being Index (WHO-5) was used to examine well-being. WHO-5 comprises five items on participants’ feelings over the last two weeks, with items rated on a 6-point Likert scale. A higher score indicated better well-being. The Filipino version of WHO-5 is available on the WHO website, and its validity were examined among Filipino workers [37]. Although WHO-5 originally aimed to examine mental well-being, it can be used as a screening tool to examine mental health conditions, such as depression [38].

World Health Organization Quality of Life - BREF (WHOQOL-BREF) was used to examine participants’ quality of life. WHOQOL-BREF was developed by WHO and is a brief version of WHOQOL-100 [39]. It assesses quality of life cross-culturally and is available in over 30 languages, including Filipino. The WHO defines quality of life as an individual's perception of their position in life in the context of the culture and value systems in which they live and in relation to their goals, expectations, standards, and concerns. It has four domains: physical health (seven instruments), psychosocial health (six instruments), social relationships (three instruments), and environment (eight instruments), in addition to overall QOL and general health (two instruments). It has 26 instruments, including reverse-scored items, with a five-point Likert scale. In each domain, excluding overall QOL and general health, the average score is calculated and it is converted 100-point scale. Higher scores indicated higher quality of life.

Moreover, depression severity (mild, moderate, or severe) at the time of examination and diagnosis, based on the categorization of DSM-5-TR, was confirmed by a psychiatrist or resident doctor who received additional training and education in mental health [32].

Data were collected with the support of local health workers. For data collection from the psychiatric unit, health workers read the questionnaire and recorded the answers. If the participants preferred to answer the questionnaire by themselves and health workers thought they had no literacy difficulties, the questionnaire was self-administered. Data were collected from the community sample using a self-administered questionnaire. Health workers received half-day training on ethical considerations and the importance of reading up the structured questionnaire without rephrasing or summarizing.

Statistical analysis

Gender and religion were compared using Pearson’s chi-square test, and family income and K6 score were analyzed using the Mann-Whitney U test. Construct validity was assessed using Pearson’s correlation between K6, PHQ-9, WHO-5, and WHOQOL-BREF. Reliability was assessed with Cronbach’s α. Furthermore, years of education were compared with K6 for their correlation. Moreover, based on clinical judgment of patients' depressive status at the time of examination, patients were divided into two groups (mild depression and moderate or severe depression), and K6 scores were compared using an unpaired t-test to assess psychometric validity. Additionally, the distribution of K6 total scores among community members, psychiatric unit patients, and patients with mild, moderate, and severe depression was used to determine the optimal cutoff points for moderate and severe psychological distress. IBM SPSS Statistics for Windows, Version 29 (Released 2023; IBM Corp., Armonk, New York, United States) was used for all analyses. Statistical significance was set at *p* < 0.05.

Ethical consideration

Before data collection, objective and process of the study, voluntariness of participation, anonymity of answers, right to withdraw from the study, and compensation were verbally explained to all potential participants using a written document. If potential participants agreed to participate in the study after the explanation, all participants, except those with literacy difficulties, provided written informed consent. People with literacy difficulties provided their finger stamp, and names and addresses were written by health workers or families in the informed consent sheet. As the result, informed consent was obtained from all individual participants included in the study.

Regarding data collection for participants from the community, ethical approval was obtained by the ethics committee of the Organization of Global Affairs, Kanazawa University (Approval Number: KINDAIKOKUKI-001GO). Data from the psychiatric unit were collected with ethical approval from the Institute of Human and Social Sciences Ethics Committee of Kanazawa University (Approval Number: 2023-79). The Muntinlupa City Health Office approved the study protocol. The study was conducted in accordance with the ethical standards as laid down in the 1964 Declaration of Helsinki and its later amendments.

## Results

Table 1 shows participants’ socio-demographic characteristics and compares participants from the psychiatric unit and the community. In total, 95 people from the psychiatric unit (27 male, 77 female, and one gender-diverse patient) and 405 people from the community (178 male, 226 female, and one gender-diverse patient) participated in the study. The mean age was 34.21 and 35.68 years for patients in the psychiatric unit and community, respectively. 82% and 100% of each group believed in Christianity, respectively. Statistical differences were observed in gender (*χ*= 12.27, *p* < 0.01), religion (*χ*= 40.80, *p* < 0.01), number of family members living together (*t* = 2.75, *p* < 0.05), years of education (*t* = -4.18, *p* < 0.01), and K6 total score (*u* = 31860.00, *p* < 0.01). Patients in the psychiatric unit had higher K6 total scores (median 14.00) than the community (median 3.00).

**Table 1:** Socio-demographic characteristics of the participants ^a^Unpaired *t*-test; ^b ^*χ*2analysis; ^c^Mann-Whitney U test, * *p* < 0.05, ** *p* < 0.01

	Patients in the psychiatric unit (n = 95)		Community people (n= 405)		*t*^a^/*χ*^b^/*U*^c^	
	Mean^a^/ Frequency^b^/Median^c^	SD^a^/ %^b^/ 1st-3rd quartile^c^	Mean^a^/ Frequency^b^/Median^c^	SD^a^/ %^b^/ 1st-3rd quartile^c^		
Gender^b^					12.27	**
Male	27.00	25.70	178.00	44.00		
Female	77.00	723.30	226.00	55.80		
Gender diverse	1.00	1.00	1.00	0.20		
Age^a^	34.21	12.19	35.68	12.60	1.02	
Religion^b^					40.80	**
Christianity	82.00	90.10	405.00	100.00		
Other than Christianity	6.00	6.60	0.00	0.00		
Don't believe in any	3.00	3.30	0.00	0.00		
Total number of family members living together ^a^	4.11	2.16	4.93	2.60	2.75	**
Education years ^a^	12.88	3.96	11.06	2.37	-4.18	**
Total score of K6^c^	14.00	9.50-18.00	3.00	0.00-8.00	31860.00	**
Severity of depression ^b^						
Mild	54	60.00				
Moderate	32	35.60				
Severe	4	4.40				

Table 2 shows the diagnosis profiles of participants in the psychiatric unit. Most participants were diagnosed with depressive disorder (n = 38, 38.78%), followed by anxiety disorder (n = 32, 32.65%), bipolar and related disorders (n = 16, 16.33%), and schizophrenia spectrum and other psychotic disorders (n = 8, 8.16%).

**Table 2:** Participants' diagnosis based on DSM-5-TR

Disorder	Frequency	%
Neurodevelopment Disorders	1	1.02
Schizophrenia Spectrum and Other Psychotic Disorders	8	8.16
Bipolar and Related Disorders	16	16.33
Depressive Disorders	38	38.78
Anxiety Disorders	32	32.65
Obsessive-Compulsive and Related Disorders	1	1.02
Trauma- and Stressor-Related Disorders	2	2.04
Dissociative Disorders	0	0.00
Somatic Symptom and Related Disorders	0	0.00
Feeding and Eating Disorders	0	0.00
Elimination Disorders	0	0.00
Sleep-Wake Disorders	0	0.00
Sexual Dysfunctions	0	0.00
Gender Dysphoria	0	0.00
Disruptive, Impulse- Control, and Conduct Disorders	0	0.00
Substance-Related and Addictive Disorders	0	0.00
Neurocognitive Disorders	1	0.93
Personality Disorders	0	0.00
Paraphilic Disorders	0	0.00
Other Mental Disorders and Additional Codes	0	0.00
Medication-Included Movement Disorders and Other Adverse Effects of Medication	0	0.00
Other Conditions That May Be a Focus of Clinical Attention	0	0.00

The correlation coefficients between K6 and PHQ-9, WHO-5, WHOQOL-BREF, and education year are listed in Table 3. The PHQ-9 score was strongly and positively correlated with the K6 score (*r* = 0.74, *p* < 0.01). The score of WHO-5 (*r* = -0.51, *p* < 0.01) and total score of WHOQOL-BREF (*r* = -0.54, *p* < 0.01), Physical health (*r* = -0.48, *p* < 0.01), Psychosocial health (*r* = -0.59, *p* < 0.01), and Social relationship (*r* = -0.40, *p* < 0.01) in the domain of WHOQOL-BREF were moderately and negatively associated with the K6 score. Environment in the domain of WHOQOL-BREF (r = -0.25) was negatively and weakly correlated with K6, whereas years of education (r = 0.08) was not significantly correlated with the K6 total score.

**Table 3:** Pearson’s correlation coefficients with the total K6 score *p < 0.05, **p < 0.01 PHQ-9: Patient Health Questionnaire-9; WHO-5: The World Health Organization-Five Well-Being Index; WHOQOLBREF: The World Health Organization Quality of Life - BREF Mean total K6 score = 12.36 (SD: 5.84)

Variables	Mean	SD	r	
PHQ-9	13.36	5.84	0.74	^**^
WHO-5	12.42	4.98	-0.51	^**^
Physical health in WHOQOL-BREF	11.95	2.24	-0.48	^**^
Psychosocial health in WHOQOL-BREF	12.55	3.07	-0.59	^**^
Social relationship in WHOQOL-BREF	13.74	3.25	-0.40	^**^
Environment in WHOQOL-BREF	13.75	2.52	-0.25	*
Education year	12.96	3.95	0.08	

Table 4 compares each K6 score and the total score between patients with mild depression and those with moderate or severe depression. Moreover, Cronbach's alpha coefficients in total and in the case where each item was deleted were calculated. Except for items 1 and 3, each K6 item and the total score were statistically different for each group. The total scores of patients with mild depression were 12.48 (SD: 5.40) and patients with moderate or severe depression were 15.60 (SD: 5.47), respectively. Cronbach’s alpha coefficient for K6 was 0.87. Except for item 1, all items contributed to Cronbach’s alpha coefficient. Although not indicated in the table, no significant difference was observed in the K6 total score between men (mean: 13.70, SD: 5.69) and women (mean: 13.13, SD: 5.89).

**Table 4:** Comparison of K6 based on the severity of depression assessed by the doctor and Cronbach's alpha coefficient Unpaired *t*-test  ^*^p < 0.05,  ^**^p < 0.01 Cronbach’s alpha coefficient for the total K6 score is 0.88

		Patients assessed as having mild depression		Patients assessed as having moderate or severe depression		t		Cronbach's alpha coefficient when the item is deleted
		Mean	SD	Mean	SD			
1	nervous?	2.19	1.21	2.61	1.08	-1.69		0.90
2	hopeless?	1.74	1.19	2.46	1.25	-2.73	^**^	0.86
3	restless or fidgety?	2.25	1.11	2.47	1.11	-0.95		0.86
4	so depressed that nothing could cheer you up?	2.00	1.15	2.56	1.16	-2.24	^*^	0.84
5	that everything was an effort?	2.07	1.30	2.86	1.15	-2.94	^**^	0.85
6	worthless?	1.93	1.26	2.56	1.34	-2.27	^*^	0.85
	Total K6 score	12.48	5.40	15.60	5.47	-2.63	^*^	

Table 5 shows the distribution of K6 scores among people from the community, patients in the psychiatric unit, patients with mild depression, and patients with moderate or severe depression; 29. 90%, 84.30%, 80.70%, and 91.40% of people in the community, patients in the psychiatric unit, patients with mild depression, and patients with moderate or severe depression, respectively, scored 7 or above. Thus, 6/7 could be the optimal cutoff point for moderate psychological distress. Moreover, the cutoff point for serious psychological distress was suggested as ≥ 12. 13.3%, 62.9%, 55.8%, and 77.1% of people in the community, patients in the psychiatric unit, patients with mild depression, and patients with moderate or severe depression, respectively scored 12 or above.

**Table 5:** Distribution of the total K6 score

	Community people		Patients in the psychiatric unit		Patients assessed as having mild depression		Patients assessed as having moderate or severe depression	
	Frequency	Cumulative percentage	Frequency	Cumulative percentage	Frequency	Cumulative percentage	Frequency	Cumulative percentage
0	130	32.1	0	0.0	0	0	0	0
1	16	36.0	0	0.0	0	0	0	0
2	53	49.1	2	2.2	2	3.5	0	0
3	16	53.1	1	3.4	1	5.3	0	0
4	26	59.5	0	3.4	0	5.3	0	0
5	11	62.2	1	4.5	0	5.3	1	2.9
6	32	70.1	10	15.7	8	19.3	2	8.6
7	11	72.8	4	20.2	3	24.6	1	11.4
8	19	77.5	1	21.3	0	24.6	0	11.4
9	12	80.5	3	24.7	2	30.8	1	14.3
10	18	84.9	5	30.3	4	38.5	1	17.1
11	7	86.7	6	37.1	3	44.2	2	22.9
12	28	93.6	5	42.7	3	50	2	28.6
13	8	95.6	3	46.1	0	50	3	37.1
14	7	97.3	7	53.9	3	55.8	4	48.6
15	2	97.8	2	56.2	2	59.6	0	48.6
16	4	98.8	8	65.2	7	73.1	1	51.4
17	3	99.5	6	71.9	3	78.8	3	60
18	1	99.8	8	80.9	6	90.4	2	65.7
19	0	99.8	4	85.4	2	94.2	2	71.4
20	1	100.0	1	86.5	1	96.2	0	71.4
21	0	100.0	5	92.1	1	98.1	4	82.9
22	0	100.0	2	94.4	0	98.1	2	88.6
23	0	100.0	3	97.8	0	98.1	3	97.1
24	0	100.0	2	100.0	1	100	1	100

The Filipino version of K6 is shown in Appendices as Table 6. 

## Discussion

This study examined the psychometric validity, construct validity, and internal consistency of K6 in clinical and community settings using a dual sample. A convenience sampling method for recruitment of patients in the psychiatric unit and a randomized sampling method for community people were employed.

Patients in the psychiatric unit had statistically higher K6 total scores (median 14.00) than the community people (median 3.00). In addition, there were statistically significant differences in gender, education, and religion. In the patients in the psychiatric unit, there were more female patients, smaller number of family members living together, fewer Christian patients, and those with longer years of education.

Cronbach’s alpha coefficient for the K6 total scores was 0.88, indicating sufficient reliability. Mean of Cronbach’s alpha coefficient on K6 in various language versions reported in the meta-analysis is 0.84, with 95% CI [0.81, 0.86] [2]. The results of this study for internal consistency are consistent with those of the previous study [2], and the current set of items on K6 is appropriate.

Moreover, this study examined the construct validity with correlations between K6, PHQ-9, WHO-5, and WHOQOL-BREF. K6 and PHQ-9 (*r* = 0.74, *p* < 0.01), as screening instruments for major depressive disorder, were strongly and positively correlated. K6 and WHO-5 (*r* = -0.51, *p* < 0.01), physical health (*r* = -0.48, *p* < 0.01), psychosocial health (*r* = -0.59, *p* < 0.01), and social relationships (*r* = -0.40, *p* < 0.01) in the domain of WHOQOL-BREF were moderately and negatively correlated. Another study on validation of the K6 reported a coefficient of r = 0.71 with the PHQ-9 [40]. Construct validity is confirmed, and its number is roughly consistent with the previous study and the expected direction.

Comparison of K6 scores based on depression severity assessed by a doctor showed that patients with moderate or severe depression had significantly higher K6 scores. This suggests that the Filipino version of K6 has strong psychometric properties based on clinical examination by a doctor and is appropriate to assess the severity of depression in the clinical setting. The developer of K6 indicated the importance of assessing severity rather than only diagnosis [10]. Given the limited availability of mental health professionals in LMICs, including the Philippines, the implementation of the K6 scale in both community and clinical settings may enhance the early identification of mental health problems, whether through primary health care providers or self-assessment, thereby facilitating timely and appropriate interventions.

Previous studies on K6 determined the cutoff points for serious and moderate physiological distress. Serious psychological distress or serious mental illness includes mental health problems causing moderate-to-serious impairment in social, occupational, or school functioning and requires treatment based on the diagnosis criteria of the Diagnostic and Statistical Manual of Mental Disorders (DSM-IV) [10,41]. Furthermore, moderate psychological distress can be characterized as the need of mental health care is more compared to people with higher psychological distress stress but less than those with serious psychological distress [42]. For instance, the cutoff point for serious psychological distress is ≥ 13 in the USA and Hong Kong and 10 in Iran, and that for moderate psychological distress is ≥ 5 in the USA [42-45]. A government document cited in The Lancet and other epidemiological studies indicated that the prevalence rate of mental disorders in the Philippines is approximately 10.5-11.6% [46-48]. 13.3% of community people were included at the cutoff point 11/12 for serious psychological distress. This number approximately corresponds to the prevalence rate of mental disorders in the Philippines. Therefore, the optimal cutoff for severe psychological distress is suggested as 11/12. In addition, a cutoff for moderate psychological distress can be suggested as 6/7. Only 8.6% of people with moderate or severe depression assessed by the doctor were missed at the cutoff point of 6/7 for moderate psychological distress lower score would promote mental health awareness in the community.

This study has several limitations. First, it did not include a diagnosis-based interview, and sensitivity and specificity based on receiver operating characteristic (ROC) analysis were not examined. The cutoff point was determined by the prevalence rate in the country and distribution of K6 scores of each group. Finally, participants were recruited from one region in Metro Manila, and socio-demographic characteristics, including gender, and the date collection period between the two groups were slightly different. Therefore, further study with populations from different regions of the Philippines and ROC analysis may be important.

However, this study employed a dual-sample (community people and patients in a psychiatric unit) and examined psychometric validity, construct validity, and internal consistency of the Filipino version of K6. As a result, the Filipino version of K6 was validated and can be used to screen mental health conditions in clinical and community settings in the Philippines. In addition, further study with a Filipino version of K6 is expected to promote mental health in not only clinical settings but also in community-based settings.

## Conclusions

This study examined psychometric validity, construct validity, and internal consistency of the Filipino version of K6 in clinical and community settings. Psychometric validity, construct validity, and internal consistency were confirmed. The cutoff points for moderate and serious psychological distress can be suggested as 6/7 and 11/12, respectively, based on the distribution of the K6 score of community people and patients in the psychiatric unit. The Filipino version of K6, including cutoff points, will be a useful tool in the local context in practice and can be used as a measurement tool in studies to promote mental health in various settings.

## Appendices

**Table 6:** Filippino version of the Kessler Psychological Distress Scale (K6)

	Ang mga sumusunod na tanong ay inaalam ang iyong mga naranasan o naramdaman sa nakaraang tatlumpung 30 araw. Pakibasa ng mabuti at bilugan ang numero ng iyong sagot. (The following questions are about how you have been feeling during the past 30 days. Please select the number that best describes how often you had this feeling.)	Hindi kailanman (None of the time)	Madalang/ manaka-naka (A little of the time)	Minsan (Some of the times)	Madalas (Most of the time)	Palagi/ Paulit-ulit (All the time)
1	…nerbiyos? (nervous?)	0	1	2	3	4
2	…kawalan ng pag-asa? (hopeless?)	0	1	2	3	4
3	…hindi ka mapalagay o hindi mapakali? (restless or fidgety?)	0	1	2	3	4
4	…depresyon o matinding kalungkutan na parang walang makakapagpasaya sa iyo? (so depressed that nothing could cheer you up?)	0	1	2	3	4
5	…pakiramdam na lahat ng ginagawa mo ay napakabigat? (that everything was an effort?)	0	1	2	3	4
6	…pakiramdam na hindi ka mahalaga o wala kang kwenta? (worthless?)	0	1	2	3	4
